# Association between SOD2 T-9C and MTHFR C677T polymorphisms and longevity: a study in Jordanian population

**DOI:** 10.1186/1471-2318-9-57

**Published:** 2009-12-15

**Authors:** Omar F Khabour, Essa S Abdelhalim, Ahmad Abu-Wardeh

**Affiliations:** 1 Department of Medical Laboratory Sciences, Jordan University of Science and Technology, Irbid 22110, Jordan

## Abstract

**Background:**

Aging in animals is associated with high body oxidative stress, which might affect susceptibility and onset of age-related diseases, and the subsequent life span. Therefore, genes that modulate oxidative stress might play a role in determining longevity. In this study, we investigated whether the gene pool relevant to the *SOD2*-9T/C and *MTHFR *677C/T polymorphisms changes as the Jordanian population ages.

**Methods:**

Polymorphisms were genotyped in 130 elderly subjects (57 females and 73 males, mean age: 90.01 years) and 135 young control subjects (67 females and 68 males, mean age: 33.43 years).

**Results:**

No significant differences were found in the genotype and allele frequencies of examined *SOD2 *and *MTHFR *gene variants between the elderly group and young controls (*P *> 0.05), nor when each gender was considered separately (*P *> 0.05).

**Conclusion:**

*SOD2*-9T/C and *MTHFR *677C/T gene polymorphisms do not seem to be important in Jordanian population for longevity phenotype.

## Background

Aging involves increases in oxidative stress status presented by elevated levels of oxidized forms of biomolecules in the body of the organism [1]. This leads to tissue damage and decreases in body functions, homeostasis, and tolerance to chronic diseases [2,3]. Thus, genes that modulate oxidative stress might play a role in human longevity.

In this study, we investigated association of *SOD2 *-9T/C SNP and *MTHFR *677 C/T SNP with longevity in Jordanian population. The *SOD2 *gene codes for the mitochondrial manganese superoxide dismutase, a major cellular antioxidative stress enzyme [4]. SOD2 dismutates the superoxide anion into hydrogen peroxide that is detoxified into water by glutathione peroxidases and catalase [5]. Presence of the C allele at -9 position of *SOD2 *gene results in substitution of alanine for valine (Ala16Val) in the mitochondrial targeting sequence [6]. This substitution partially retains SOD2 enzyme within the narrow inner membrane import pore and lowers the enzyme activity [7,8]. The importance of *SOD2 *-9T/C polymorphism is indicated by its association with several age related diseases such as cancer [9,10] and diabetic nephropathy [11].

The *MTHFR *gene codes for methylenetetrahydrofolate reductase that catalyzes the conversion of 5,10-methylenetetrahydrofolate to 5-methyltetrahydrofolate. The latter serves as a methyl donor in the reaction converting homocysteine to methionine [12]. The T allele at 677 position of *MTHFR *gene causes substitution of alanine to valine and the resulting decreases in enzyme activity and increases in body homocysteine concentrations [13]. Excess homocysteine undergoes auto-oxidation in plasma, so that free oxygen radicals are produced thereby enhancing endothelial tissue damage and inflammation [14]. In addition, excess homocysteine can directly impair DNA methylation, resulting in altered gene expression [15]. The *MTHFR *677 C/T polymorphism has been shown to be associated with ischemic stroke [16], cancer [17] and coronary artery disease [18].

Previous reports are variable on the association between *SOD2 *-9 SNP or *MTHFR *677 SNP with human longevity. For example, positive association has been reported between *SOD2 *-9 SNP and Ashkenazi males [19], but not Italian population [20]. Similarly, positive association has been reported between *MTHFR *SNP, and Swiss population or Ashkenazi women [19,21], but not Irish population [22]. In this study, we report absence of association between -9 T/C *SOD2 *SNP or 677 C/T *MTHFR *SNP with longevity in the examined sample of Jordanian elderly.

## Methods

One hundred thirty unrelated elderly subjects (> 85 years, mean age 90.01 year) volunteered from different parts of Jordan to take part in this study. Another 135 unrelated young control subjects (range from 20 to 50 years, mean age 33.34 years) were matched long-lived individuals for geographical origin. Subjects with cardiovascular diseases, diabetes, or cognitive impairments were excluded from the study. The experimental design and the sample size were similar to most longevity studies reviewed by Glatt et al., [23]. Subject's mean ages were selected based on the mortality rate in the Jordanian population, which is approximately constant from childhood to late forties, thereafter, it starts gradually inclining to reach maximum in late seventies [24]. Therefore, individuals who reach more than 85 years are rare in Jordan. An official identification document was required to participate in the study. Acceptable documents include civil ID card, birth certificate, family book, passport and military card, otherwise enrolment in the study was denied. All subjects received written and verbal explanation of the study before giving consent. The study protocol was approved by the Institutional Review Boards of Jordan University of Science and Technology.

Blood samples (1-3 ml) in EDTA tubes were obtained from all subjects. DNA was extracted from all samples using Wizard DNA Extraction Kit (Promega, Madison, USA) according to the manufacturer instructions. DNA samples were stored at -20°C until used. The concentration of the extracted DNA was measured using SmartSpect™ 3000 (Bio-Rad, Hertfordshire, UK).

*SOD2 *T-9C polymorphism was typed using RFLP-PCR protocol. Briefly, 20 μl reaction mixture containing 5 ng of template DNA, 0.75 unit GoTaq polymerase (Promega, Madison, USA), and a final concentration of 200 mM each deoxynucleotide and 1× reaction buffer, and 1 mM of forward (5'-ACC AGC AGG CAG CTG GCG CCG G-3') and reverse (5'-GCG TTG ATG TGA GGT TCC AG-3') primers. Cycling was performed at 95°C for 15 min and 35 cycles at 94°C for 30 s, 65°C for 30 s and 72°C for 30 s, followed by a final extension of 7 min at 72°C. PCR products were detected using electrophoresis on 4% agarose, confirming the presence of a 107 bp product. The *NgoMIV *enzyme (Fermentas. GmbH, St. Leon-Rot, Germany) digestion was carried out in 20 ul reaction mixture containing 3 units of enzyme and 10 μl of PCR product at 37°C for 4 hours. Materials from individuals homozygous for *SOD2 *-9 T allele don't cut with *Ngo*MIV and remain as a 107 bp product. The homozygous *SOD2 *-9 C allele cuts with *Ngo*MIV to give 89 bp and 18 bp fragments.

The *MTHFR *C677T polymorphism was also analyzed by PCR-RFLP using *Hinf *I enzyme (Fermentas). PCR primers were: forward primer (5'-TGA AGG AGA AGG TGT CTG CGG GA-3') and reverse primer (5'-AGG ACG GTG CGG TGA GAG TG-3'). Polymerase chain reaction and *Hinf *I digestion condition were similar to that described for SOD2 -9 SNP except for the annealing temperature, which was 60°C in this case. PCR fragments from *MTHFR *677 C allele don't cut with *Hinf *I and remain as a 198 bp product while fragments from *MTHFR *677 T allele cut with *Hinf *I to give 175 bp and 23 bp fragments.

The genotype distributions of the examined polymorphisms were analyzed in agreement with Hardy-Weinberg equilibrium. To test association between longevity and the polymorphic loci, distributions of allele and genotype frequencies were compared between young and elderly groups using the chi-square and Fisher's exact tests. The test power was calculated for alleles frequency using Power and Sample Size Calculation Program (PS version 3.0.1, Vanderbilt University Medical Center, Nashville, TN, USA) and for genotype frequencies using SAS macro [25]. For all analysis, the power was more than 75%. The SPSS 15.0 statistical software package (SPSS Inc., Chicago, IL) was used for statistical analysis. *P values *smaller than *0.05 *were considered significant.

## Results

Jordan is a small country located in Southwest Asia and classified among the low income countries. The population is predominantly Arab (98%) and most of it is urban (70%) [26]. According to the 2007 census, the total population of Jordan was 5.7 million, percentage of individuals of 65 years of age or older was 4.1% and life expectancy at birth in the total population was 73 year [26].

The average age of the elderly group in the study was 90.01 years. In Jordan, the mortality rate starts inclining exponentially at fifty year-old getting maximum level in the late seventies indicating that reaching above 85 year-old is a rarity (Khoury et al., 1999). Therefore, oldest old people (> 85 year-old) are considered exceptional individuals in Jordan.

Males to females ratio was 1.3:1 in the elderly group and 1:1 in the control group (*P = 0.346*). Number of relatives who exceeded 85 year-old was higher in the elderly group compared to the young control group (70.3% versus 59.8%, respectively, *P = 0.013*). The higher number of relatives who exceeded 85 year-old (>25%) in the elderly group indicates the presence of genetic component to longevity in the Jordanian population.

Table 1 shows the frequency of homozygous and heterozygous genotypes for *SOD2 *T-9C and *MTHFR *C677T SNPs in our sample of elderly and young controls. The genotype frequencies of the *SOD2 *T-9C SNP of elderly and control groups were not statistically different (Chi square test, *P = 0.576*). Accordingly, the frequency of SNP -9 T to C was not significantly different between elderly and controls (Chi square test, *P = 0.355*). Similar results were observed with the *MTHFR *C677T SNP (Chi square test: for genotype frequencies, *P = 0.944 *and for allele frequencies, *P = 0.727*).

**Table 1:** Frequencies of SOD2 and MTHFR alleles and genotypes in elderly and control groups.

Genotypes and Alleles	Control group N (percentage)	Elderly group N (percentage)	P value
-9 SOD2 *			
TT	42 (31.1)	44 (33.8)	
TC	61 (45.2)	62 (47.7)	
CC	32 (23.7)	24 (18.5)	0.576
Allele T	145 (53.7)	150 (57.7)	
Allele C	125 (46.3)	110 (42.3)	0.355
			
677 MTHFR *			
CC	82 (60.7)	77 (59.2)	
CT	41 (30.4)	40 (30.8)	
TT	12 (8.9)	13 (10.0)	0.944
Allele C	205 (75.9)	194 (74.6)	
Allele T	65 (24.1)	66 (25.4)	0.727

* All groups were in Hardy Weinberg equilibrium (P > 0.05).

Several studies indicated that gender was a main variable in the genetics of longevity and suggested that men and women might follow different pathways to reach longevity [27,28]. In our sample, genotypes and alleles frequencies for examined SNPs were not different when males were considered alone (Chi square test: for -9 T/C *SOD2*, *P = 0.691 *and for 677 C/T *MTHFR*, *P = 0.795*, Table 2), or when females were considered alone (Chi square test: for -9 T/C SOD2, *P = 0.317 *and for 677 C/T MTHFR, *P = 0.792*, Table 3).

**Table 2:** Frequencies of SOD2 and MTHFR alleles and genotypes in elderly and control male subjects

Genotypes and Alleles	Control males group N (percentage)	Elderly males group N (percentage)	P value
**-9 SOD2**			
TT	17 (25)	23 (31.5)	
TC	35 (51.5)	34 (46.6)	
CC	16 (23.5)	16 (21.9)	0. 691
Allele T	69 (50.7)	80 (54.8)	
Allele C	67 (49.3)	66 (45.2)	0.495
			
**677 MTHFR**			
CC	42 (61.8)	41 (56.2)	
CT	21 (30.9)	26 (35.6)	
TT	5 (7.4)	6 (8.2)	0.795
Allele C	105 (77.2)	108 (74.0)	
Allele T	31 (22.8)	38 (26.0)	0.528

**Table 3:** Frequencies of SOD2 and MTHFR alleles and genotypes in elderly and control female subjects

Genotypes and Alleles	Control Females group N (percentage)	Elderly Females group N (percentage)	P value
**-9 SOD2**			
TT	25 (37.3)	21 (36.8)	
TC	26 (38.8)	28 (49.1)	
CC	16 (23.9)	8 (14.0)	0. 317
Allele T	76 (56.7)	70 (61.4)	
Allele C	58 (43.3)	44 (38.6)	0.455
			
**677 MTHFR**			
CC	40 (59.7)	36 (63.2)	
CT	20 (29.9)	14 (24.6)	
TT	7 (10.4)	7 (12.3)	0.792
Allele C	100 (74.6)	73 (72.3)	
Allele T	34 (25.4)	28 (27.7)	0.686

## Discussion

Oxidative stress is a condition where the redox balance between oxidant and antioxidant is shifted toward an oxidized state. In animals, oxidative stress increase with ageing due to high production of free radicals by aged mitochondria and decreased cellular antioxidant capacity. The mitochondrial magnesium superoxide dismutase (SOD2) is considered the first line of defense against reactive oxygen species [4]. The gene for *SOD2 *has a common T to C polymorphism, resulting in a valine to alanine change at the 16 position of its mitochondrial targeting sequence (Ala16Val), which affects the structure of the protein [6], and reduces its entrance into the mitochondria [29] leading to increased oxidative stress.

The *MTHFR *gene also affects oxidative stress status in human body. The gene codes for an enzyme that play a key role in the folate metabolism [30]. Nucleotide transition (C to T) at nucleotide 677 of *MTHFR *causes alanine to valine substitution in the N-terminal catalytic domain, leading to 30% and 65% reduction in activity for heterozygotes and homozygotes of the variant allele, respectively [31]. Reduced activity of MTHFR leads to high levels of blood homocysteine, which is rapidly auto-oxidized, leading to the production of cytotoxic reactive oxygen species and to endothelial damage [32].

In this study, we hypothesized that the presence of the C allele at -9 position of *SOD2 *and T allele at position 677 of *MTHFR *might decrease life span. The data showed no statistically significant difference between the elderly and young groups when comparing genotypic distributions and allelic frequencies of studied *SOD2 *and *MTHFR *polymorphisms (Table 1). In agreement with our results, De Benedictis et al., [20] showed that *SOD2 *variant does not affect individual life expectancy in Italian population (sample size: 109, age criterion > 100 years old). In addition, Brattstrom et al., [22] reported that *MTHFR *C677T allele is not a strong risk factor for premature death in Ireland (sample size: 1388, age criterion > 80 years old). In animal models, mice deficient in SOD2 (Sod2-/-) exhibit neonatal lethality in association with dilated cardiomyopathy and a massive lipid accumulation in the liver [33], while (Sod2+/-) heterozygous mice have increased cancer incidence without affecting aging [34]. Furthermore, SOD isoforms showed no effect on life span in *C. elegans *[35] and Drosophila [36]. In contrast to our results, positive association has been reported in Danish population (sample size: 1650, age criterion > 92 years old) [37] and Ashkenazi males (sample size 150, > 75 years old [19] with *SOD2 *-9 SNP, while for *MTHFR*, positive association has been reported in Swiss population (sample size: 104, age criterion > 65 years old) and Ashkenazi women (sample size: 74, age criterion > 75 years old) [19,21]. The discrepancy in the finding of the different studies might be due to difference in experimental design, sample size and criteria used in selecting subjects. In addition, the examined polymorphisms/longevity associations might have a population specific component, being affected by the population specific gene pool as well as by gene-environment interaction.

Among the limitations of this study are the sample size and age of recruitments of elderly subjects (≥ 85 years). One hundred and thirty subjects with a mean age of 90.01 years were included in the present study. The population of Jordan was 5.7 million in 2007 and only 4.1% of the total population was individuals of 65 years of age or older [26]. In addition, the mortality rate starts inclining exponentially at fifty year-old getting maximum level in the late seventies indicating that reaching above 85 year-old is a rarity (Khoury et al., 1999). Therefore, oldest old people are considered exceptional individuals in Jordan. Moreover, the lack of elderly centers in Jordan makes it very hard to recruit elderly subjects that fit sampling criteria. Despite all these obstacles, the sample size of the current research fall within the range of longevity studies reviewed by Glatt et al., [23] and previous studies that asked the same question in other populations (see discussion above). Future studies with a bigger sample size might be more appropriate with this kind of research.

Longevity is a complex trait, which likely results from a blessed combination of genetic and non-genetic factors [38]. It is possible that the excess of environmental factors exert stronger influence on longevity than the genetic traits [39]. For example, in institutionalized or home-bound elderly, oxidative stress was reported to increase significantly [40,41], while in free living elderly it is not always elevated [42]. In addition, *MTHFR *C677T polymorphism effect on homocysteine level can be minimized by folate intake. Studies attempting to assess the overall genetic influence on variations in the human life span indicated that approximately a quarter of the variation in the adult life spans could be attributed to genetic variation among individuals [43]. Thus, the strong influence of environmental factors on longevity might wipe the most likely weaker effect of genetic factors as observed in this study. However, the result which shows that number of relatives who exceeded 85 year-old was higher in the elderly group by approximately 25% compared to the young control group indicates the presence of genetic component to longevity in the Jordanian population. It is possible that other polymorphisms are present in the region of the examined genes in the Jordanian population; this might buffer out or modulate the effect of the studied loci. Therefore, further studies are required to screen for the presence of such modifier polymorphisms in addition to direct measurement of levels and activity of gene products of the examined loci in according to subject's genetic background.

Reaching extreme age without diseases is one aspect of successful ageing [23]. In this study, elderly individuals with cardiovascular diseases, diabetes, or cognitive impairments were excluded from the current study. Previous studies have shown that *SOD2 *-9T/C and *MTHFR *677 C/T polymorphisms were associated with diabetes, cancer and cardiovascular diseases in other populations [9,10]. Thus, these polymorphisms might also associate with certain diseases in the Jordanian population. Exploring this possibility is a matter of future research.

## Conclusion

In this study, we investigated the contribution of the *SOD2*-9T/C and *MTHFR *677C/T gene polymorphisms to the longevity phenotype in the Jordanian population. The results of this study indicate that *SOD *-9T/C and *MTHFR *677C/T are not important determinant of life span in Jordanian population.

## Competing interests

The authors declare that they have no competing interests.

## Authors' contributions

OK designed the study, supervised molecular experiments, analyzed data and prepared manuscript. EA conducted genotyping experiments, performed statistical analysis and participated in recruitment of subjects. AA participated in recruitment of subjects and blood sampling.

## Pre-publication history

The pre-publication history for this paper can be accessed here:

http://www.biomedcentral.com/1471-2318/9/57/prepub
